# Radar-Based Control of a Helical Microswimmer in 3-Dimensional Space with Dynamic Obstacles

**DOI:** 10.34133/cbsystems.0158

**Published:** 2025-06-02

**Authors:** Yuezhen Liu, Yibin Wang, Kaiwen Fang, Hui Chen, Guangjun Zeng, Jiangfan Yu

**Affiliations:** ^1^ School of Science and Engineering, The Chinese University of Hong Kong, Shenzhen 518172, China.; ^2^ Shenzhen Institute of Artificial Intelligence and Robotics for Society (AIRS), Shenzhen 518129, China.

## Abstract

Advanced control strategies critical for microrobots have been widely investigated to achieve precise locomotion. However, dynamic obstacle avoidance in 3D space is a major challenge in control that remains unsolved. In this work, a control scheme is developed for the automatic navigation of a helical microswimmer in 3-dimensional (3D) space with dynamic obstacles. A 3D hierarchical radar with a motion sphere and a detection sphere is firstly developed. Using the radar-based avoidance approach, the desired motion direction for the microswimmer to avoid obstacles can be obtained, and the coarse-to-fine search is used to decrease the computational load of the algorithm. Three navigation modes of the microswimmer in 3D space with dynamic conditions are realized by the radar-based navigation strategy that combines the global path planning algorithm and the radar-based avoidance approach. Subsequently, a motion controller is proposed to achieve precise 3D locomotion control of the microswimmer. The control scheme integrating the radar-based navigation strategy and the motion controller is developed. The experimental results of navigated locomotion of a helical microswimmer in 3D space with 8 static obstacles and 8 dynamic obstacles demonstrate the effectiveness of the control scheme, and the proposed control scheme paves the way for advanced locomotion control of helical microswimmers in complex 3D space.

## Introduction

Magnetic microrobots have attracted considerable attention recently due to their great potential in biomedical applications, such as targeted drug delivery, biosensing, and embolization [1–8]. Various magnetic microrobots have been investigated, such as miniature soft robots [9–13], bio-hybrid microrobots [14,15], and microswarms [16–18]. Helical microswimmers have been widely used, since they can achieve efficient and precise locomotion under a low-strength rotating magnetic field in complex or physiological environments [19,20]. To date, helical microswimmers have played an important role in magnetic manipulation [21], in vitro transportation of DNA [22], and drug transportation in vivo [23].

Advanced control strategies are critical for helical microswimmers to avoid obstacles with precise locomotion. A microswimmer can follow a predefined path to reach the target while avoiding collisions with static obstacles, using a model-free control method with image-based visual servoing [24]. A control strategy based on informed RRT* and sliding mode control is developed for the navigation of a microswimmer in an environment with multiple static obstacles [25]. The optimal bidirectional RRT* is used to generate a collision-free path to navigate a microswimmer to the targeted position in 3-dimensional (3D) space [26]. These methods are applied in static environments, and the control strategies used for the navigation of microrobots in environments with dynamic obstacles have also been investigated. A deep learning-based control method is proposed for microswarms composed of nanoparticles to avoid dynamic obstacles automatically [27]. A 2D radar-based control strategy is developed for a vortex-like microswarm to avoid multitype dynamic obstacles in a micromaze [28]. The aforementioned methods are developed for the navigation of microrobots in 2D space with dynamic obstacles. However, these methods have challenges to be applied in 3D space with dynamic obstacles, due to high computational load of path planning in 3D space. Therefore, control scheme for microrobots to achieve dynamic obstacle avoidance in 3D space with a high updating frequency of motion direction is demanded, especially in complex 3D space with multiple dynamic and static obstacles.

This work proposes a 3D radar-based control scheme that realizes the navigated locomotion of microswimmers in 3D space with multiple static and dynamic obstacles. We firstly introduce the kinematic model of the helical microswimmer. The radar-based avoidance approach is subsequently developed for the microswimmer to avoid dynamic obstacles in 3D space, and the coarse-to-fine search is used to decrease the computational load of the algorithm. A radar-based navigation strategy is proposed by combining global path planning algorithm and the radar-based avoidance approach. A motion controller that contains a neural network-based feedforward controller and a fuzzy logic controller (FLC) is proposed for the precise 3D locomotion control of the microswimmer. The radar-based control scheme that combines the radar-based navigation strategy and the motion controller is proposed. The simulation and experimental results of navigated locomotion of the microswimmer in 3D space with 8 static and 8 dynamic obstacles demonstrate the effectiveness of the radar-based control scheme.

The contributions of this article include the following. (a) A radar-based avoidance approach is developed for the helical microswimmer to avoid dynamic obstacles in 3D space with a high updating frequency of the motion direction. (b) A radar-based navigation strategy combing radar-based avoidance approach and the global path planning algorithm is proposed for the automatic navigation of the helical microswimmer in 3D space with multiple static and dynamic obstacles. (c) The radar-based control scheme integrating the motion controller and the radar-based navigation strategy is proposed, and the helical microswimmer can perform navigated locomotion while avoiding collisions with multiple static and dynamic obstacles in 3D space using the control scheme.

## Methods

### Models

**Fig. 1.**
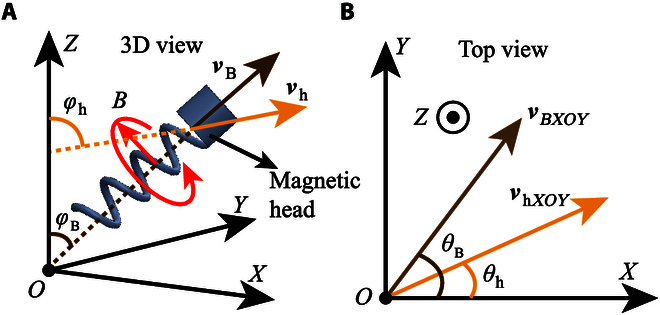
The schematics in (A) 3D view and (B) top view of a helical microswimmer actuated by a rotating magnetic field. The rotating magnetic field *B* is denoted by the red arrow. The brown and orange arrows represent the propulsion direction and the motion direction of the helical microswimmer, respectively.

A helical microswimmer with a magnetic head can be actuated by a rotating magnetic field [29], and the schematics is shown in Fig. 1. The rotating magnetic field is homogeneous and can be expressed as:

$$\begin{array}{c} \begin{array}{ll} B & ={\left[B_{x}B_{y}B_{z}\right]}^{T}\\ & =A\;\left[\begin{array}{c} -\text{cos}\left(\varphi_{B}\right)\;\text{cos}\left(\theta_{B}\right)\;\text{cos}\left(2\pi \text{ft}\right)-\text{sin}\left(\theta_{B}\right)\;\text{sin}\left(2\pi \text{ft}\right)\\ \text{cos}\left(\varphi_{B}\right)\;\text{sin}\left(\theta_{B}\right)\;\text{cos}\left(2\pi \text{ft}\right)-\text{cos}\left(\theta_{B}\right)\;\text{sin}\left(2\pi \text{ft}\right)\\ \text{sin}\left(\varphi_{B}\right)\;\text{cos}\left(2\pi \text{ft}\right) \end{array}\right] \end{array} \end{array}$$ (1)

where A is the amplitude of the magnetic field and f represents the frequency of the field. The propulsion direction of the helical microswimmer $v_{B}$ is characterized by the pitch angle $\varphi_{B}$ and the direction angle $\theta_{B}$, which are the angle of the propulsion direction with respect to the *z* axis, and the angle of the projection of the propulsion direction at the horizontal *xoy* plane with respect to the *x* axis, respectively. Similarly, the motion direction of the helical microswimmer $v_{h}$ is determined by $\varphi_{h}$ and $\theta_{h}$. Due to disturbances, including the induced drifting and the gravity of the helical microswimmer, $v_{h}$ is not aligned with $v_{B}$ [30].

When the amplitude A and the frequency f of the magnetic field remain constant, and f is below the step-out frequency $f_{\text{step}}$, the velocity of the helical microswimmer is only affected by its geometry [31], and the kinematic model of the microswimmer is:

$$\left\{\begin{array}{c} {\overset{\cdot }{x}}_{h}\left(t\right)=\mathrm{cf}\text{sin}\left(\varphi \left(t\right)\right)\text{cos}\left(\theta \left(t\right)\right)\\ {\overset{\cdot }{y}}_{h}\left(t\right)=\mathrm{cf}\text{sin}\left(\varphi \left(t\right)\right)\text{sin}\left(\theta \left(t\right)\right)\\ {\overset{\cdot }{z}}_{h}\left(t\right)=\mathrm{cf}\text{cos}\left(\varphi \left(t\right)\right) \end{array}\right.$$ (2)

where c is a positive constant decided by the geometry of the microswimmer, and $P_{h}\left(t\right)={\left[x_{h}\left(t\right),y_{h}\left(t\right),\;z_{h}\left(t\right)\right]}^{T}$ is the real-time position of the helical microswimmer.

### Radar-Based Navigation Strategy

We firstly develop a radar-based avoidance approach for the helical microswimmer to avoid dynamic obstacles in 3D space. A radar-based navigation strategy that combines the radar-based avoidance approach and the global path planning algorithm is subsequently proposed for the automatic navigation of the helical microswimmer in 3D space with multiple static and dynamic obstacles.

#### Selection of desired motion direction

**Fig. 2.**
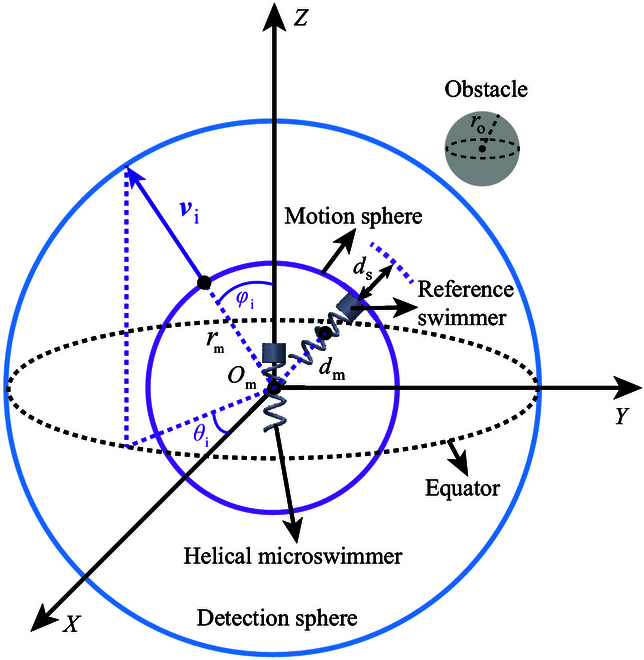
The schematics of the 3D hierarchical radar.

The key element of the radar-based avoidance approach is the 3D hierarchical radar. The schematics of the 3D hierarchical radar is shown in Fig. 2. The 3D hierarchical radar is composed of a motion sphere (i.e., the purple sphere) and a detection sphere (i.e., the blue sphere). The detection sphere is used for obstacle detection. If no obstacle enters the detection sphere, the desired motion direction of the helical microswimmer $v_{\mathrm{de}}={\left[\theta_{\mathrm{de}}\left(t\right),\varphi_{\mathrm{de}}\left(t\right)\right]}^{T}$ will point from the microswimmer toward the goal position, which can be expressed as:

$$\left\{\begin{array}{c} \;\theta_{\mathrm{de}}\left(t\right)=\text{arctan}\frac{y_{g}-y_{h}\left(t\right)}{x_{g}-x_{h}\left(t\right)}\\ \varphi_{\mathrm{de}}\left(t\right)=\text{arccos}\frac{z_{g}-z_{h}\left(t\right)}{\text{Dist}\left(P_{g},\;P_{h}\left(t\right)\right)} \end{array}\right.$$ (3)

where $P_{g}={\left[x_{g},\;y_{g},\;z_{g}\right]}^{T}$ is the position of the goal.

If there exists obstacle entering the detection sphere, the avoidance maneuver will be activated. In this case, the desired motion direction of the microswimmer will be selected from optional motion directions. The optional motion directions are generated by dividing all directions around the microswimmer in 3D space uniformly, and the ith optional motion direction is denoted by $v_{i}={\left[\theta_{i},\varphi_{i}\right]}^{T}$, as shown by the purple arrow in Fig. 2. We define reference swimmers for direction selection. The reference swimmer indicates the position of the current helical microswimmer when it moves distance $d_{m}$ along one of the optional motion directions. The position of the current helical microswimmer is $P_{h}\left(t\right)={\left[x_{h}\left(t\right),\;y_{h}\left(t\right),\;z_{h}\left(t\right)\right]}^{T}$, and the position of the ith reference swimmer is $P_{\mathrm{rh}}\left(i,\;t\right)={\left[x_{\mathrm{rh}}\left(i,\;t\right),y_{\mathrm{rh}}\left(i,\;t\right),z_{\mathrm{rh}}\left(i,\;t\right)\right]}^{T}$. In this case, the ith optional motion direction for the helical microswimmer can be expressed as:

$$\left\{\begin{array}{c} \;\theta_{i}\left(t\right)=\text{arctan}\frac{y_{\mathrm{rh}}\left(i,\;t\right)-y_{h}\left(t\right)}{x_{\mathrm{rh}}\left(i,\;t\right)-x_{h}\left(t\right)}\\ \varphi_{i}\left(t\right)=\text{arccos}\frac{z_{\mathrm{rh}}\left(i,\;t\right)-z_{h}\left(t\right)}{\text{Dist}\left(P_{\mathrm{rh}}\left(i,\;t\right),\;P_{h}\left(t\right)\right)} \end{array}\right.$$ (4)

All optional motion directions for the helical microswimmer can then be described using the following equation:

$$\begin{array}{c} \left\{{\left[\theta_{i}\left(t\right),\;\varphi_{i}\left(t\right)\right]}^{T}\left|\begin{array}{c} \theta_{i}\left(t\right)=\text{arctan}\frac{y_{\mathrm{rh}}\left(i,\;t\right)-y_{h}\left(t\right)}{x_{\mathrm{rh}}\left(t,\;i\right)-x_{h}\left(t\right)},\\ \Delta \theta_{i}=\Delta \theta ,\;i=1,2,\ldots ,\frac{360\times 180}{\Delta \theta \cdot \Delta \varphi }\\ \varphi_{i}\left(t\right)=\text{arccos}\frac{z_{\mathrm{rh}}\left(i,\;t\right)-z_{h}\left(t\right)}{\text{Dist}\left(P_{\mathrm{rh}}\left(i,\;t\right),\;P_{h}\left(t\right)\right)},\\ \Delta \varphi_{i}=\Delta \varphi ,\;i=1,2,\ldots ,\frac{360\times 180}{\Delta \theta \cdot \Delta \varphi } \end{array}\right.\right\} \end{array}$$ (5)

where Δθ and Δφ are the angular resolutions of the direction angle θ and the pitch angle φ, respectively. Based on Eq. (5), each optional motion direction corresponds to a reference swimmer, and the motion sphere circumscribes all reference swimmers, as shown by the purple sphere in Fig. 2.

The selection of the desired motion direction during avoidance maneuver $v_{\mathrm{de}}={\left[\theta_{\mathrm{de}}\left(t\right),\;\varphi_{\mathrm{de}}\left(t\right)\right]}^{T}$ is based on an objective function and a selection constraint. The objective function can be expressed as:

$$\begin{array}{c} \begin{array}{l} \sigma_{i}=k_{g}N\left(\frac{1}{\text{Dist}\left(P_{\mathrm{rh}}\left(i,t\right),\;P_{g}\right)}\right)\\ \;+k_{a}N\left(\text{Dist}\left(P_{\mathrm{rh}}\right.\left(i,t\right),\;\left.P_{o}\right)-\frac{l_{h}}{2}-r_{o}\right) \end{array} \end{array}$$ (6)

where $l_{h}$ is the length of the helical microswimmer and $P_{o}$ is the position of the obstacle. The radius of the obstacle is denoted by $r_{o}$, as shown in Fig. 2. The function $k_{g}N\left(\frac{1}{\text{Dist}\left(P_{\mathrm{rh}}\left(i,\;t\right),\;P_{g}\right)}\right)$ indicates the propensity of the ith optional motion direction to point toward the goal position, and the function $k_{a}N\left(\text{Dist}\left(P_{\mathrm{rh}}\right.\left(i,\;t\right),\left.P_{o}\right)-\frac{l_{h}}{2}-r_{o}\right)$ represents the propensity of the ith optional motion direction to evade the obstacle. $N\left(\cdot \right)$ represents the normalization. The positive coefficients $k_{g}$ and $k_{a}$ represent the weights of the two propensities, respectively. The value of $k_{g}$ and $k_{a}$ are controlled based on the following equation:

$$\begin{array}{c} \left[\begin{array}{c} k_{g}\\ k_{a} \end{array}\right]=\left\{\begin{array}{c} \left[\begin{array}{c} k_{s}\\ 1 \end{array}\right],\text{only static obstacles exist}\\ \left[\begin{array}{l} 1\\ 0 \end{array}\right]+A_{d}\left[\begin{array}{l} K_{g}\\ K_{a} \end{array}\right],\text{dynamic obstacles exist} \end{array}\right. \end{array}$$ (7)

where $k_{s}$ is a positive constant that determines $k_{g}/k_{a}$ when only static obstacles enter the detection sphere. When dynamic obstacles exist, $K_{g}$ and $K_{a}$ are set based on the artificial potential field approach [32], as shown by the following equation:

$$\begin{array}{c} \;K_{g}=\frac{1}{2}\mu_{\mathrm{at}}\text{Dist}{\left(P_{h}\left(t\right),\;P_{g}\right)}^{2}\\ K_{a}=\frac{1}{2}\mu_{\mathrm{re}}{\left(\frac{1}{\text{Dist}\left(P_{h}\left(t\right),\;P_{o}\right)}-\frac{1}{r_{\mathrm{de}}}\right)}^{2} \end{array}$$ (8)

where $\mu_{\mathrm{at}}$ and $\mu_{\mathrm{re}}$ are positive coefficients of attraction and repulsion, respectively. The radius of the detection sphere is $r_{\mathrm{de}}$. In Eq. (7), $A_{d}$ is the matrix to control the range of $k_{g}$ and $k_{a}$, which can be expressed as:

$$A_{d}=\left[\begin{array}{cc} \frac{\alpha }{\frac{1}{2}{r_{\mathrm{de}}}^{2}} & 0\\ 0 & \frac{\beta }{{\left(\frac{1}{\frac{l_{h}}{2}+r_{o}}-\frac{1}{r_{\mathrm{de}}}\right)}^{2}} \end{array}\right]$$ (9)

where positive coefficients α and β are used to keep $k_{g}$ larger than $k_{a}$. Based on Eqs. (7) to (9), the weights of the two propensities $k_{g}$ and $k_{a}$ can be automatically tuned. In Eq. (6), $\sigma_{i}$ denotes the quality of the ith optional motion direction. The desired motion direction during the avoidance maneuver has the maximum $\sigma_{i}$, i.e., $\sigma_{\max}$. The position of the corresponding reference swimmer is $\begin{array}{c} P_{\mathrm{rh}}\left(\text{max},t\right)={\left[x_{\mathrm{rh}}\left(\text{max},t\right),y_{\mathrm{rh}}\left(\text{max},t\right),z_{\mathrm{rh}}\left(\text{max},t\right)\right]}^{T} \end{array}$, and the desired motion direction $v_{\mathrm{de}}={\left[\theta_{\mathrm{de}}\left(t\right),\varphi_{\mathrm{de}}\left(t\right)\right]}^{T}$ can be expressed as:

$$\begin{array}{c} \left\{\begin{array}{c} \theta_{\mathrm{de}}\left(t\right)=\text{arctan}\frac{y_{\mathrm{rh}}\left(\text{max},\;t\right)-y_{h}\left(t\right)}{x_{\mathrm{rh}}\left(\text{max},\;t\right)-x_{h}\left(t\right)}\\ \varphi_{\mathrm{de}}\left(t\right)=\text{arccos}\frac{z_{\mathrm{rh}}\left(\text{max},\;t\right)-z_{h}\left(t\right)}{\text{Dist}\left(P_{\mathrm{rh}}\left(\text{max},\;t\right),\;P_{h}\left(t\right)\right)} \end{array}\right. \end{array}$$ (10)

The selection constraint can be expressed as:

$$\text{Dist}\left(P_{\mathrm{rh}}\left(i,t\right),P_{o}\right)-\frac{l_{h}}{2}-r_{o}>d_{s}$$ (11)

where $d_{s}$ is the safety distance, and we use $\begin{array}{c} \text{Dist}\left(P_{\mathrm{rh}}\left(i,t\right),P_{o}\right)\;-\;\frac{l_{h}}{2}\;-\;r_{o} \end{array}$ to represent the distance between the reference swimmer and the obstacle, as a rational simplification. Based on Eq. (11), if the distances between the reference swimmers and the obstacle are shorter than $d_{s}$, the corresponding optional motion directions will be regarded as unsafe directions that are excluded from the selection of the desired motion direction.

#### Coarse-to-fine search

The desired motion direction $v_{\mathrm{de}}$ during obstacle avoidance is selected from optional motion directions shown in Eq. (5), and the computational load is decided by the number of the optional motion directions. Based on Eq. (5), the number of the optional motion directions num can be expressed as $\mathrm{num}=\frac{360\times 180}{\Delta \theta \cdot \Delta \varphi }$, where Δθ and Δφ are the angular resolution of the direction angle θ and the pitch angle φ, respectively. If Δθ and Δφ are set small, e.g., Δθ = Δφ = 1 degree, the number of the optional motion directions will be 180 × 360, which demands a high computational resource to obtain the desired motion direction. In contrast, large Δθ and Δφ, e.g., Δθ = Δφ = 30 degree, decrease the number of the optional motion directions (i.e., num=12×6). Although the computational load is low in this case, the desired motion direction selected from a small number of optional motion directions will be coarse to avoid the obstacle, which increases the risk of collisions and may lead to a longer travelling path. To balance the high precision and low computational load, we adopt the coarse-to-fine search for the selection of the desired motion direction in 3D space. The coarse-to-fine search has two stages, i.e., coarse search and fine search. The optional motion directions in the coarse-to-fine search can be expressed as:

$$\begin{array}{c} \begin{array}{c} \text{Coarse search}:\left\{\begin{array}{c} -180\leq \theta_{i}\leq 180,i=1,2,\ldots ,\frac{360\times 180}{\Delta \theta_{1}\cdot \Delta \varphi_{1}}\\ 0\leq \varphi_{i}\leq 180,i=1,2,\ldots ,\frac{360\times 180}{\Delta \theta_{1}\;\cdot \;\Delta \varphi_{1}} \end{array}\right.\\ \;\text{Fine search}:\left\{\begin{array}{c} \theta_{\mathrm{de}1}\left(t\right)-\Delta \theta_{f}\leq \theta_{i}\leq \theta_{\mathrm{de}1}\left(t\right)+\Delta \theta_{f},\\ i=1,2,\ldots ,\frac{2\Delta \theta_{f}\;\cdot \;2\Delta \varphi_{f}}{\Delta \theta_{2}\;\cdot \;\Delta \varphi_{2}}\\ \varphi_{\mathrm{de}1}\left(t\right)-\Delta \varphi_{f}\leq \varphi_{i}\leq \varphi_{\mathrm{de}1}\left(t\right)+\Delta \varphi_{f},\\ i=1,2,\ldots ,\frac{2\Delta \theta_{f}\;\cdot \;2\Delta \varphi_{f}}{\Delta \theta_{2}\cdot \Delta \varphi_{2}} \end{array}\right. \end{array} \end{array}$$ (12)

where $\Delta \theta_{1}$ and $\Delta \varphi_{1}$ are angular resolutions in the coarse search, and $\Delta \theta_{1}$ and $\Delta \varphi_{1}$ are set large to decrease the computational load. The desired motion direction selected in the coarse search is $v_{\mathrm{de}1}={\left[\theta_{\mathrm{de}1}\left(t\right),\;\varphi_{\mathrm{de}1}\left(t\right)\right]}^{T}$, and the optional motion directions in the fine search are around $v_{\mathrm{de}1}$. The angular resolutions in the fine search are $\Delta \theta_{2}$ and $\Delta \varphi_{2}$, which are set small to ensure the precision. The $\Delta \theta_{f}$ and $\Delta \varphi_{f}$ are used to determine the ranges of optional motion directions in the fine search. The desired motion direction selected in the fine search is $v_{\mathrm{de}2}$, and it is also the final desired motion direction $v_{\mathrm{de}}$ obtained by the radar-based avoidance approach.

The coarse-to-fine search is realized based on the characteristics of the 3D hierarchical radar and can ensure high precision and low computational load simultaneously, which shows the improvment of the radar-based avoidance approach compared to other conventional algorithms such as RRT.

#### Global path planning integrated with radar-based avoidance

**Fig. 3.**
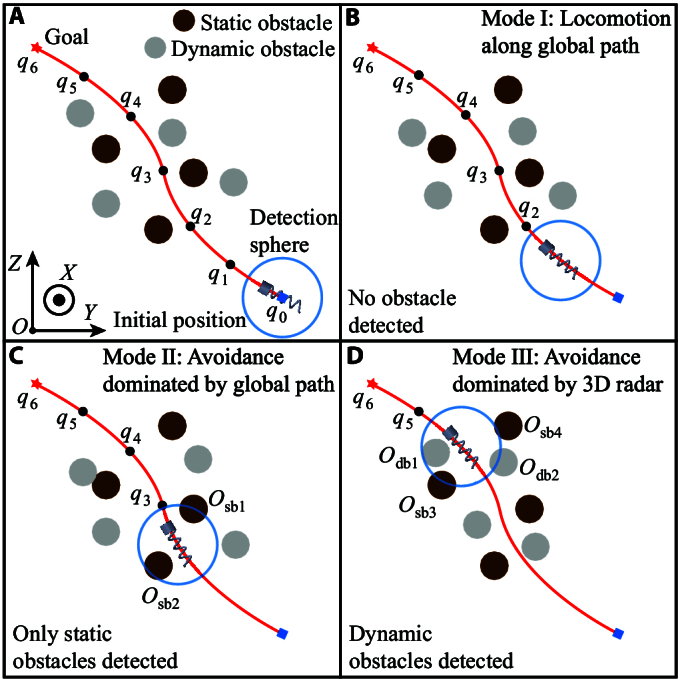
The schematics of the combination of the global path planning algorithm and the radar-based avoidance approach for automatic navigation of a helical microswimmer. Key points of the global path are denoted by $q_{0},q_{1},\ldots ,q_{6}$. (A) Schematics of the initial global path planning. (B to D) Schematics of navigation modes I to III.

Herein, we combine the global path planning algorithm and the radar-based avoidance approach for the navigated locomotion of the microswimmer in 3D space with multiple dynamic and static obstacles, and the schematics are shown in Fig. 3. The global path planning algorithm is firstly implemented to generate a global reference trajectory for the navigation of microswimmer from the initial position to the goal position while avoiding collisions with static obstacles, as shown in Fig. 3A. The key points of the global reference path (i.e., $q_{0},q_{1},\ldots ,q_{6}$) are phased targets to guide the microswimmer. Subsequently, the radar-based avoidance approach is used for the microswimmer to avoid dynamic obstacles in 3D space.

Three navigation modes can be realized based on the combination of the global path planning algorithm and the radar-based avoidance approach, as shown from Fig. 3B to D. In mode 1, no static and dynamic obstacles enter the detection sphere (i.e., the blue circle) of the 3D radar. In this case, the avoidance maneuver is not activated, and the microswimmer will move toward the phased target (i.e., $q_{2}$ in Fig. 3B) along the global reference path. In mode 2, as shown in Fig. 3C, only static obstacles (i.e., $O_{\mathrm{sb}1}$ and $O_{\mathrm{sb}2}$) enter the detection sphere. The avoidance maneuver is activated, and the desired motion direction of the microswimmer is selected from optional motion directions in Eq. (5). In mode 2, $k_{g}/k_{a}$ in the objective function in Eq. (6) is set to a constant based on Eq. (7), in order to decrease the computational load caused by the change of $k_{g}$ and $k_{a}$. In this case, the key point acting as the phased target in the global path dominates the avoidance maneuver, and the desired motion direction is mainly determined by the selection constraint in Eq. (11). In mode 3, dynamic obstacles enter the detection sphere, as shown by $O_{\mathrm{db}1}$ and $O_{\mathrm{db}2}$ in Fig. 3D. In this case, $k_{a}$ dynamically increases when the distance between the obstacle and the microswimmer is smaller, which further decreases the risk of collisions in dynamic environments. In the three navigation modes, the key point that acts as the phased target for the microswimmer is determined by the following iteration model:

$$\begin{array}{c} \left\{\begin{array}{c} \begin{array}{c} \;Q\left(t\right)=\left\{q_{j}\;\left|j=m,\;m+1,\ldots \right.\;,m+k,\;\text{Dist}\left(q_{i},\;P_{h}\left(t\right)\right)<M_{q}\;\right\} \end{array}\\ \;q_{p}\left(i\right)=q_{m+k}\in Q\left(t\right),\exists \text{Dist}\left(q_{i},\;P_{h}\left(t\right)\right)<M_{q}\\ \;q_{p}\left(i\right)=q_{p}\left(i-1\right),\forall \text{Dist}\left(q_{i},\;P_{h}\left(t\right)\right)\geq M_{q} \end{array}\right. \end{array}$$ (13)

In Eq. (13), $Q\left(t\right)$ is the set of key points with distances less than $M_{q}$ from the position of the helical microswimmer $P_{h}\left(t\right)$, and $M_{q}$ is a positive constant to control the number of key points in $Q\left(t\right)$. The last point in $Q\left(t\right)$ (i.e., $q_{m+k}$) is selected as the phased target $q_{p}\left(i\right)$ for the microswimmer in the ith iteration, when there exist key points with distances less than $M_{q}$ from $P_{h}\left(t\right)$. If there exist no key points with distances less than $M_{q}$ from $P_{h}\left(t\right)$, the phased target $q_{p}\left(i\right)$ will be the same as that in the previous iteration, i.e., $q_{p}\left(i-1\right)$.

### Control Methodology

Using the rotating magnetic field, the helical microswimmer can perform locomotion by propulsion, and the propulsion direction is $v_{B}$. However, due to disturbances, the actual motion direction of the helical microswimmer $v_{h}$ could deviate from $v_{B}$. In this section, we firstly propose a feedforward controller based on radial basis function (RBF)–extreme learning machine (ELM) neural network to compensate the direction deviation between $v_{B}$ and $v_{h}$. An FLC is then designed as the feedback controller to further tune $v_{B}$ to decrease the deviation between $v_{h}$ and the desired motion direction $v_{\mathrm{de}}$.

In the feedforward controller, the relationship between the motion direction $v_{h}={\left[\theta_{h},\varphi_{h}\right]}^{T}$ and the propulsion direction $v_{B}={\left[\theta_{B},\varphi_{B}\right]}^{T}$ is modeled by RBF-ELM networks. The relationship can be expressed as:

$$F\left({\left[\theta_{h},\varphi_{h}\right]}^{T}\right)={\left[\theta_{B},\varphi_{B}\right]}^{T}$$ (14)

The input and the output of the network are $X_{i}={\left[\theta_{hi},\varphi_{hi}\right]}^{T}$ and $Y_{i}={\left[\theta_{Bi},\varphi_{Bi}\right]}^{T}$, respectively. The RBF-ELM network contains three functional layers, i.e., the input layer, the hidden layer, and the output layer. The activation function in the hidden layer of the RBF-ELM network is the RBF. The RBF $R_{1},R_{2},\ldots ,R_{m}$ can be expressed as:

$$R_{m}=e^{-\left(w_{m}X_{i}+b_{m}\right)}$$ (15)

The weight $w_{m}$ and the bias $b_{m}$ in the hidden layer of the RBF-ELM network are initialized randomly and remained fixed, which is the characteristic of the ELM network. It simplifies the learning process to make the network faster to train than traditional multi-layer perceptron (MLP) neural networks [33].

Based on the model trained from the RBF-ELM network, i.e., $F\left(\cdot \right)$ in Eq. (14), the predefined control input $v_{\mathrm{BP}}$ is obtained, which can be expressed as:

$$\begin{array}{c} v_{\mathrm{BP}}={\left[\theta_{\mathrm{BP}},\varphi_{\mathrm{BP}}\right]}^{T}=F\left({\left[\theta_{\mathrm{de}},\varphi_{\mathrm{de}}\right]}^{T}\right)=F\left(v_{\mathrm{de}}\right) \end{array}$$ (16)

Subsequently, a FLC is designed as the feedback controller to further decrease the error between the actual motion direction of the helical microswimmer $v_{h}$ and the desired motion direction $v_{\mathrm{de}}$.

**Fig. 4.**
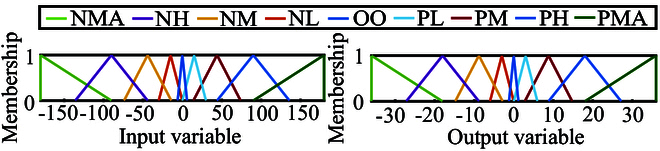
The membership functions in the FLC. NMA, negative maximum; NH, negative high; NM, negative medium; NL, negative low; OO, zero; PL, positive low; PM, positive medium; PH, positive high; PMA, positive maximum.

The input of the FLC is the direction deviation between the desired motion direction $v_{\mathrm{de}}$ and the motion direction $v_{h}$ of the helical microswimmer $\Delta v_{\mathrm{de}}$. The output of the FLC is the control variable $\Delta v_{\text{c}}$, and the sum of $\Delta v_{\text{c}}$ and the predefined control input $v_{\mathrm{BP}}$ is the final control input $v_{c}$. The membership functions of the input and the output are shown in Fig. 4. Based on the fuzzy rules and the membership functions, the control variable $\Delta v_{c}$ is obtained. Subsequently, the final control input $v_{c}$ is obtained by adding $\Delta v_{c}$ with $v_{\mathrm{BP}}$, i.e., $v_{c}=v_{\mathrm{BP}}+\Delta v_{c}$.

**Fig. 5.**
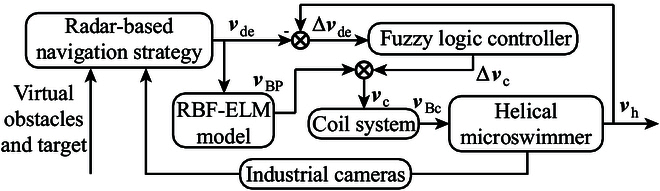
The control diagram for automatic navigation of helical microswimmers in 3D space with multiple static and dynamic obstacles.

The control diagram for automatic navigation of a helical microswimmer in 3D space with multiple dynamic and static obstacles is shown in Fig. 5. The desired motion direction $v_{\mathrm{de}}$ is firstly obtained using the radar-based navigation strategy. Subsequently, $v_{\mathrm{de}}$ is input to the motion controller to calculate the control input $v_{c}$. The motion controller consists of an RBF-ELM model used as the feedforward controller, and a FLC for feedback control. Finally, $v_{c}$ is input to the coil system to generate the rotating magnetic field with direction $v_{\mathrm{Bc}}$, and the helical microswimmer can perform desired locomotion with motion direction $v_{h}$ to avoid obstacles under the magnetic field. The industrial cameras are used to capture the position and size of the helical microswimmer for real-time vision feedback in the whole process.

### Simulation

**Fig. 6.**
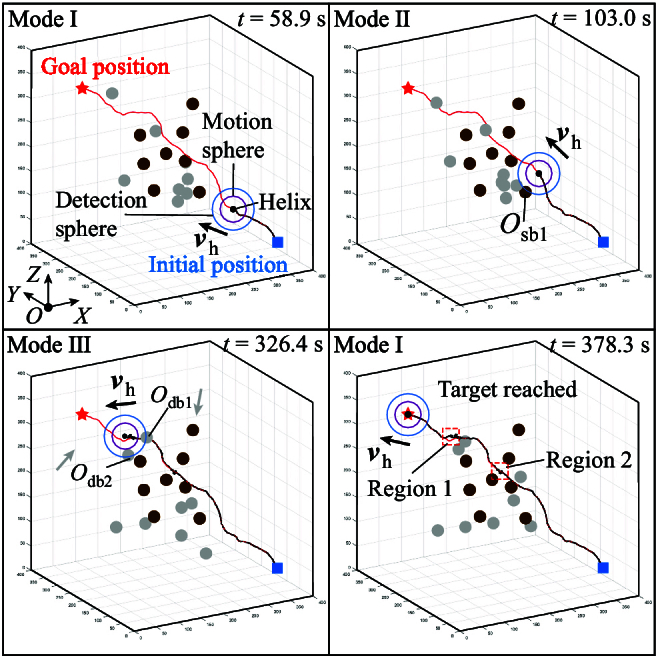
The simulation results of navigated locomotion of a helical microswimmer in 3D space with multiple static and dynamic obstacles. The brown and gray circles indicate static obstacles and dynamic obstacles, respectively. The gray arrows represent the moving directions of dynamic obstacles. The reference trajectory and moving trajectory of the microswimmer are denoted by red and black lines, respectively.

The simulation of navigated locomotion of a helical microswimmer in 3D space with multiple static and dynamic obstacles is conducted, as shown in Fig. 6. Dynamic obstacles are moving randomly, as shown by the gray circles. A global path is firstly generated from the initial position to the goal position using RRT*-connect algorithm [26] while avoiding static obstacles. The microswimmer starts moving from the initial position along the global path, and the navigation mode is 1, as shown at *t* = 58.9 s. At *t* = 103 s, a static obstacle (i.e., $O_{\mathrm{sb}1}$) enters the detection sphere, and the microswimmer performs the avoidance task with navigation mode 2. Two dynamic obstacles (i.e., $O_{\mathrm{db}1}$ and $O_{\mathrm{db}2}$) enter the detection sphere at *t* = 326.4 s, and the microswimmer will avoid the nearest obstacle to it by turn with navigation mode 3. At *t* = 378.3 s, the microswimmer reaches the target. The moving trajectory of the microswimmer differs from the reference trajectory due to avoidance of obstacles, and the microswimmer will move back to the reference trajectory after avoidance maneuver, as shown by regions 1 and 2 in Fig. 6. The simulation results validate the effectiveness of our proposed control scheme.

## Results

### The experimental setup

**Fig. 7.**
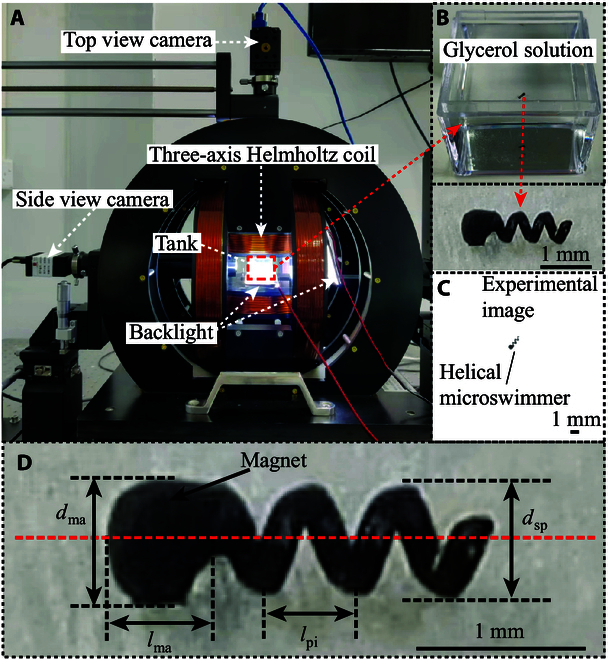
The experimental system. (A) Coil system. (B) Tank and helical microswimmer. (C) Real experimental image. (D) Helical microswimmer with detailed shape parameters. The red dashed line indicates the body axis of the helical microswimmer.

The experimental system is shown in Fig. 7. The system mainly consists of a 3D Helmholtz coil to generate the rotating magnetic field, a host computer to apply the radar-based control scheme, and two industrial cameras to capture the top and side views in the experiments for vision feedback. The helical microswimmer with detailed shape parameters is shown in Fig. 7D. The helical microswimmer is 3D printed, and a Nd-Fe-B permanent magnet is planted in its head for magnetic actuation. The length of the magnet $l_{\mathrm{ma}}$ is 500 μm, and the diameter of the magnet $d_{\mathrm{ma}}$ is 600 μm. The magnetization direction of the magnet is perpendicular to the body axis of the helical microswimmer. The body of the helical microswimmer is in the shape of spiral, as shown in Fig. 7D. The spiral is defined using diameter, pitch, and turn. The diameter $d_{\mathrm{sp}}$ is 500 μm, the length of pitch $l_{\mathrm{pi}}$ is 500 μm, and the number of turns is 3.5. The total length of the helical microswimmer $l_{h}$ is 2.25 mm. In the experiments, the helical microswimmer is emerged in glycerol with a viscosity of 950 cP. The strength and frequency of the rotating magnetic field are set to 3 mT and 7 Hz, respectively. Using the magnetic field, the microswimmer can move upward vertically with a velocity of 180 μm/s. In all experimental results, the top view and side view of the helical microswimmer are captured by a top camera and a side camera. Robot trajectories, reference trajectories, and obstacles are virtually overlaid on the real experimental image. The 3D view is generated from the real-time position data of the helical microswimmer collected from the top view and the side view.

### Trajectory tracking in 3D space

**Fig. 8.**
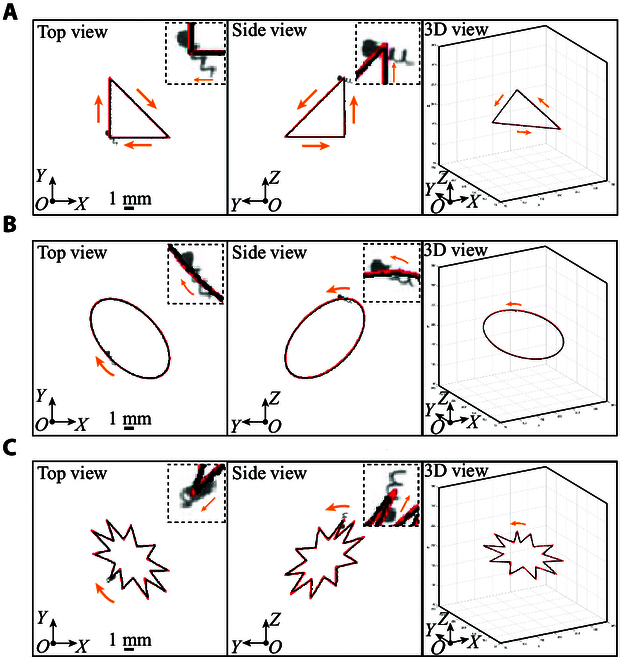
The experimental results of trajectory tracking using a helical microswimmer in 3D space. The experimental images of the helical microswimmer are enlarged in the insets. (A to C) Experimental results of triangular, circular, and star-like trajectory tracking. The red and black lines indicate the reference trajectory and moving trajectory of the microswimmer, respectively. The orange arrows denote the motion direction of the helical microswimmer. Scale bar, 1 mm.

**Fig. 9.**
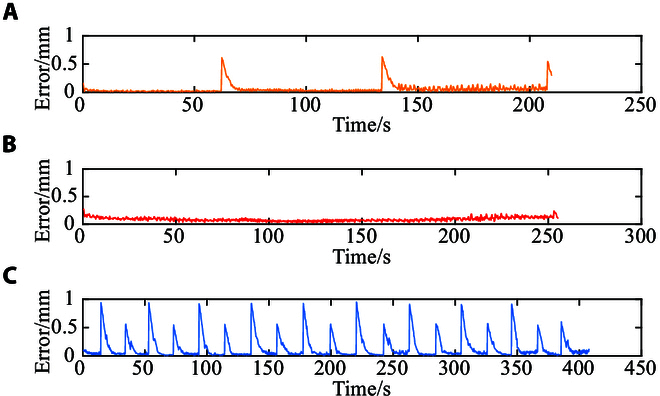
The distance error of trajectory tracking in 3D space. (A to C) Distance errors during triangular, circular, and star-like trajectory tracking.

To validate the effectiveness of the motion control strategy, the real helical microswimmer is controlled to track different trajectories in 3D space. The experimental results of triangular, circular, and star-like trajectory tracking in 3D space are shown in Fig. 8A to C, respectively. The moving trajectories of the helical microswimmer match with the reference trajectories, validating the effectiveness of the designed motion controller. The distance errors of tracking the three trajectories using the microswimmer are shown in Fig. 9A to C, respectively. The average distance errors during triangular, circular, and star-like trajectory tracking are 57.5, 87.3, and 145.1 μm, respectively. The small distance errors validate the precision of our control method.

### Automatic navigation in 3D space with multiple static and dynamic obstacles

**Fig. 10.**
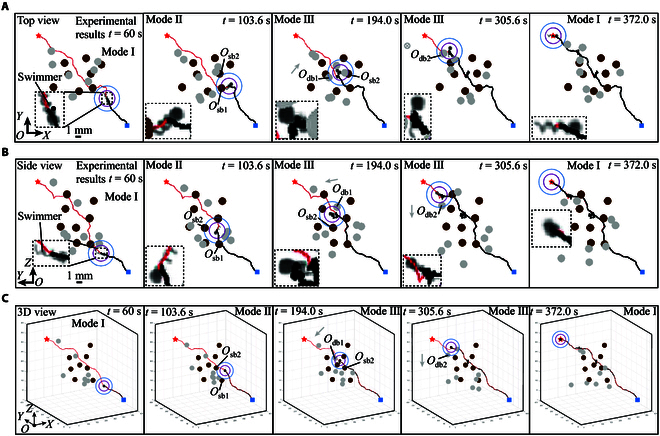
The experimental results of automatic navigation of a helical microswimmer in 3D space with multiple static and dynamic obstacles. The experimental images of the helical microswimmer are enlarged in the insets. (A to C) Experimental results in the top view, side view, and 3D view. The initial and goal positions are denoted by blue square and red star, respectively. The reference trajectory and the moving trajectory of the microswimmer are represented by red and black lines, respectively. The moving directions of dynamic obstacles are indicated by gray arrows. Scale bar, is 1mm.

The experiments of navigated locomotion of the helical microswimmer in 3D space with 8 static obstacles and 8 dynamic obstacles are conducted to validate the effectiveness of the radar-based control scheme, and the experimental results are shown in Fig. 10. The top and side views of the experimental results are shown in Fig. 10A and B, respectively, and the composite image in 3D view is shown in Fig. 10C. The static obstacles and dynamic obstacles are represented by brown circles and gray circles, respectively. The diameters of the static and dynamic obstacles are both 1.3 mm. The speeds of the dynamic obstacles range from 100 to 125 μm/s. The helical microswimmer in 3D view is denoted by the black dot.

Firstly, a global reference path is generated from the initial position (i.e., the blue square) to the goal position (i.e., the red star) using RRT*-connect algorithm for the avoidance of static obstacles [26], and the path is smoothed based on Gaussian function for smooth navigation. The smoothed global reference path is represented by red lines in Fig. 10. Subsequently, three navigation modes of the helical microswimmer are realized based on the combination of the global path planning and the radar-based avoidance approach, as mentioned in Radar-based navigation strategy. At the beginning, the microswimmer will move along the global reference path from the initial position (i.e., the blue square), and the navigation mode is 1, as shown at *t* = 60 s. At *t* = 103.6 s, two static obstacles (i.e., $O_{\mathrm{sb}1}$ and $O_{\mathrm{sb}2}$) are detected by the detection sphere of the 3D radar. Because only static obstacles are detected, the navigation mode is 2, and the motion direction of the microswimmer is selected mainly based on the selection constraint in Eq. (11). In this case, $k_{g}/k_{a}$ in the objective function is set as a constant, and the key point acting as the phased target in the global path dominates the avoidance maneuver. At *t* = 194.0 s, a static obstacle and a dynamic obstacle (i.e., $O_{\mathrm{sb}2}$ and $O_{\mathrm{db}1}$) are detected. Because there exists dynamic obstacle detected by the detection sphere, the navigation mode comes to 3. In this case, $k_{g}/k_{a}$ dynamically changes with the distance between the microswimmer and the obstacle, and the motion direction of the microswimmer is determined based on the objective function, the selection constraint, and the coarse-to-fine search. At *t* = 305.6 s, a dynamic obstacle (i.e., $O_{\mathrm{db}2}$) is detected by the detection sphere, and the navigation mode is also 3. After the avoidance maneuver, the microswimmer will move toward the key point determined by the iteration model in Eq. (13). Finally, the microswimmer reaches the goal position (i.e., the red star) with navigation mode 1 at *t* = 372 s.

The proposed control scheme is applied successfully to navigate the helical microswimmer in 3D space with multiple static and dynamic obstacles automatically, and the key point determined by the iteration model in Eq. (13) acts as the phased target for the microswimmer. Based on the phased targets and the motion controller, the influence of the disturbances on the motion of the microswimmer can be decreased, and the direction deviation between the desired motion direction and actual motion direction of the microswimmer is small, as shown by the trajectory tracking results in Fig. 10. In the whole process, the desired motion direction of the microswimmer can be updated with a frequency of 2.6 Hz, which validates the effectiveness of the proposed control scheme in dynamic environments with multiple static and dynamic obstacles.

## Conclusion

This work proposed a radar-based control scheme to automatically navigate helical microswimmers in 3D space with dynamic obstacles. The 3D hierarchical radar consisting of a motion sphere and a detection sphere is firstly developed. Using the radar-based avoidance approach, the desired motion direction for obstacle avoidance using the microswimmer can be obtained. The combination of the global path planning algorithm and the radar-based avoidance approach achieves three navigation modes of the helical microswimmer in 3D space with multiple static and dynamic obstacles. The radar-based control scheme that combines the radar-based navigation strategy and the motion controller is proposed. Based on the control scheme, the microswimmer can perform navigated locomotion in 3D space with 8 static obstacles and 8 dynamic obstacles successfully. This work focuses on the control of microrobots in 3D space with dynamic obstacles and provides an effective method for the automatic navigation of microrobots in complicated 3D space. The radar-based control scheme also has potential to be applied in targeted drug delivery using microswimmers in dynamic in vivo environments, with proper imaging modalities such as photoacoustic imaging systems. In the future, the helical microswimmer will be navigated in a large 3D workspace with dynamic obstacles using the radar-based control scheme.

## Data Availability

All data are available in the main text or the Supplementary Materials.
